# Conductive Polymer Thin Films for Energy Storage and Conversion: Supercapacitors, Batteries, and Solar Cells

**DOI:** 10.3390/polym17172346

**Published:** 2025-08-29

**Authors:** Rashid Dallaev

**Affiliations:** Department of Physics, Faculty of Electrical Engineering and Communication, Brno University of Technology, Technická 2848/8, 61600 Brno, Czech Republic; rashid.dallaev@vut.cz

**Keywords:** conductive polymers, thin films, supercapacitors, batteries, solar cells, polyaniline (PANI), polypyrrole (PPy), PEDOT:PSS, energy storage, energy conversion, electrochemical devices, flexible electronics, nanocomposites, polymer synthesis, film fabrication techniques

## Abstract

Conductive polymer thin films have emerged as a versatile class of materials with immense potential in energy storage and conversion technologies due to their unique combination of electrical conductivity, mechanical flexibility, and tunable physicochemical properties. This review comprehensively explores the role of conductive polymer thin films in three critical energy applications: supercapacitors, batteries, and solar cells. The paper examines key polymers such as polyaniline (PANI), polypyrrole (PPy), and poly(3,4-ethylenedioxythiophene) (PEDOT), focusing on their synthesis techniques, structural modifications, and integration strategies to enhance device performance. Recent advances in film fabrication methods, including solution processing, electrochemical deposition, and layer-by-layer assembly, are discussed with regard to achieving optimized morphology, conductivity, and electrochemical stability. Furthermore, the review highlights current challenges such as scalability, long-term durability, and interfacial compatibility, while outlining future directions for the development of high-performance, sustainable energy systems based on conductive polymer thin films.

## 1. Introduction

Sustainable energy technologies demand new lightweight, flexible, and cost-effective materials for efficient power generation and storage [1]. In response to climate and resource challenges, researchers are developing advanced materials for solar cells, batteries, and supercapacitors that are both high-performance and eco-friendly [2,3]. Among these, conductive polymers have emerged as a promising class, uniquely combining metal-like electronic conductivity with the ease of processing and mechanical advantages of plastics [4]. These intrinsically conductive polymers (ICPs) are organic polymers with conjugated π-electron backbones that can be doped to achieve high conductivity [5]. Early work by Shahid et al. in 2025 demonstrated conductive polyacetylene, inaugurating the field of conducting polymers; this breakthrough ultimately earned a Nobel Prize in 2000 for “the discovery and development of conductive polymers” [4]. Thus, over the past few decades, CPs have been intensively studied for electronics and energy applications.

**Figure 1:**
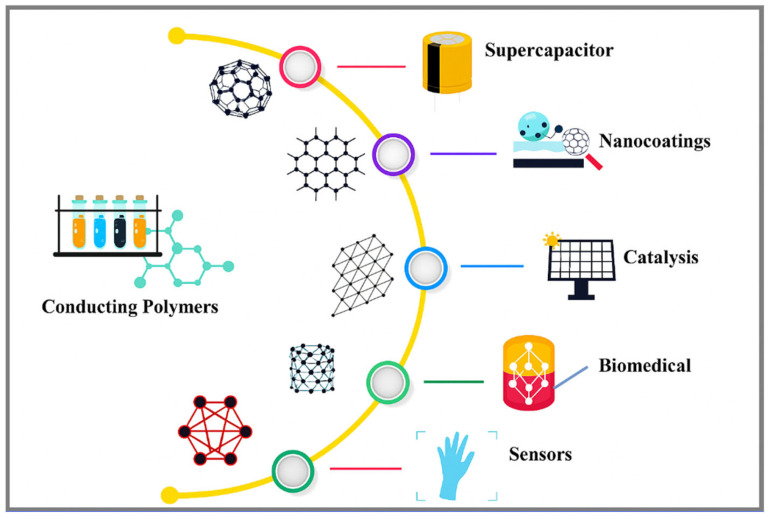
Various applications of conductive polymers. Reproduced from [17]. (The figure is available Open Access).

Conductive polymers offer a unique blend of properties that make them ideal for flexible energy devices. They conduct electricity much like metals while remaining lightweight, flexible, and easy to process as polymers [6]. Notably, CPs store charge through rapid and reversible redox reactions (pseudocapacitance), enabling very high capacitance values in electrochemical systems [7]. In effect, these materials inherit the electrical features of metals or semiconductors together with the low cost, tunable chemistry, and mechanical softness of polymers [8,9]. They have been shown to be suitable for a wide range of energy devices, including supercapacitors, batteries, and solar cells, as well as sensors and flexible electronics [10,11,12]. For example, CPs have been incorporated into supercapacitor electrodes to achieve very fast charge/discharge (high power density) and high capacitance [13,14]. Likewise, in batteries, they can serve as conductive additives or active electrode components to improve rate capability and flexibility. In organic photovoltaics, polymers such as PEDOT:PSS (poly(3,4-ethylenedioxythiophene) doped with polystyrenesulfonate) function as high-transparency electrodes or hole-transport layers, demonstrating their versatility [15,16]. Figure 1 illustrates various applications of conductive polymers.

However, in this review, the discussion shall be focused on three particular areas:

(1) Supercapacitors (Electrochemical Capacitors): CPs act as pseudocapacitive electrode materials, providing rapid surface redox reactions in addition to double-layer charge storage. Films of PANI, PPy or PEDOT with high surface area can achieve very high capacitance densities [18]. For example, hybrid electrodes combining nanostructured metal oxides with PANI have yielded transparent flexible supercapacitors with high capacitance [19,20]. Notably, PEDOT:PSS thin-film electrodes can be directly used as both current collectors and active materials in transparent all-solid-state supercapacitors [21,22]. This simplicity (spin-coating PEDOT:PSS on PET) has enabled transparent devices with tens of percent transmittance and millifarads per square centimeter capacitance. In general, the exceptional conductivity and pseudocapacitance of CPs make them ideal for supercapacitors, as extensively reviewed in the recent literature [23,24,25].
(2) Batteries: In rechargeable batteries (e.g., lithium-ion), CP thin films serve multiple purposes. They can be used as conductive additives or binders that improve electronic connectivity and mechanical flexibility of electrodes. CP coatings can also buffer volume change in high-capacity materials (such as Si) and provide continuous electron pathways. Furthermore, CPs themselves can act as redox-active cathode or anode materials in novel architectures. Reviews note that CP electrodes combine high conductivity and flexibility, although understanding their long-term cycling and ion transport is an ongoing challenge [26,27,28]. Overall, CPs have been integrated into Li-ion and Na-ion battery electrodes to enhance rate performance and durability [29,30].
(3) Solar Cells: Conductive polymer films play key roles in various solar cell technologies. In organic and perovskite solar cells (OPVs and PSCs), PEDOT:PSS films are widely used as transparent, high-work-function anodes or hole-transport layers. These films (typically ~100 nm thick) transmit >85% of visible light while maintaining good conductivity [31,32]. Thanks to this, flexible “all-plastic” solar cells have been demonstrated with PEDOT:PSS electrodes, achieving power conversion efficiencies above 12%. The excellent processability of PEDOT:PSS allows large-area, roll-to-roll fabrication of flexible solar modules. Thus, CP thin films enable lightweight, bendable solar cells by replacing brittle ITO/metal electrodes with polymeric conductors [33,34,35].

Despite their advantages, conductive polymer films still face challenges before widespread deployment in energy systems. Cycling stability is often an issue: many CPs undergo swelling, degradation or loss of conductivity under repeated redox cycling [36]. For instance, PANI and PPy electrodes can suffer from volumetric changes that reduce capacitance over time. Electrical/ionic conductivity in the film bulk also limits rate capability unless carefully engineered [37]. Moreover, the scalability and reproducibility of film fabrication must be improved for commercial devices. Achieving high mechanical durability and robust interfaces (to substrates or other materials) remains critical. For example, creating large-area CP films with uniform thickness and performance is non-trivial. Finally, long-term environmental stability (e.g., under humidity or UV light) must be addressed for outdoor applications [38,39,40].

Conductive polymer thin films combine conductive and polymeric attributes in ways that are uniquely suited for flexible energy devices [41]. By tailoring composition (copolymers, blends, dopants) and nanoarchitecture (nanowires, composites), researchers are continuously improving CP film performance. Ongoing advances in polymer chemistry and processing-such as advanced multilayer assembly and hybrid nanocomposites expected to further enhance conductivity and durability. In the coming years, these developments may enable high-performance, sustainable energy systems that leverage CP thin films for supercapacitors, batteries, and solar cells, as explored in this review [42,43].

While the field of energy conversion and storage spans a broad range of technologies, including fuel cells, thermoelectric devices, and hydrogen production, this review focuses specifically on supercapacitors, batteries, and solar cells. The rationale for selecting these three categories lies in their shared operational synergy with conductive polymer thin films:

(1) All three rely on efficient electrochemical or optoelectronic charge transport processes that can be directly enhanced by the electrical conductivity, tunable work function, and mechanical flexibility of CPs;

(2) These device types are among the most actively researched and commercially relevant platforms for lightweight, flexible, and portable energy technologies; 

(3) The body of literature on CP thin films in these applications is mature enough to allow for a comprehensive, comparative analysis, while still leaving scope for identifying performance gaps and future opportunities.

By narrowing the scope to these interrelated areas, the review provides a focused and representative overview of the current state and future potential of CP thin films in next-generation energy systems.

## 2. Definition and Classification of Conductive Polymers

Conductive polymers are organic macromolecules whose backbones support mobile charge carriers and, in many cases, exhibit metal-like electronic conductivity once “doped.” In intrinsically conducting polymers (ICPs), such as polyacetylene, polyaniline (PANI), polypyrrole (PPy), polythiophenes and PEDOT-π, conjugation along the chain enables delocalization of charge; chemical or electrochemical doping creates polarons/bipolarons that dramatically increase conductivity (from ~10^−9^–10^−7^ S cm^−1^ to ≳10–10^3^ S cm^−1^ depending on system and treatment). The microscopic picture of doping, carrier formation, and transport in conjugated polymers is now well established [10,44,45].

Performance in energy storage/conversion depends not only on the specific polymer but on how charges (electrons/ions) move through the film, the doping strategy, morphology, and whether conductivity arises from the polymer itself or from a composite network. The following complementary schemes are widely used:

1. By origin of conductivity

- Intrinsically conducting polymers (ICPs)—conductivity arises from a conjugated backbone that is modulated by doping. Representative families: PANI (proton-doped emeraldine), PPy (p-doped), polythiophenes/PEDOT, polyacetylene, donor–acceptor copolymers [10,44,46].
- Conductive polymer composites (CPCs)—insulating or weakly conducting polymers rendered conductive by adding percolating fillers (e.g., carbon black, CNTs, graphene, MXenes, metal nanowires) or by coating porous/insulating scaffolds with a thin ICP layer. Electrical transport is governed by percolation and interfacial contact [43,47,48,49].

2. By type of charge transport

- Electronic conductors—carriers are electrons/holes along the conjugated backbone (typical for dry, highly doped ICPs) [44,46].
- Ionic conductors—ion motion dominates (e.g., redox-active polymer gels/electrolytes).
- Mixed ionic–electronic conductors (OMIECs)—materials (notably PEDOT:PSS and related formulations) that support both electronic and ionic transport in swollen states, central to bioelectronics and electrochemical energy devices where ion flux couples to redox [50,51].

3. By doping chemistry and mechanism

- p-type (oxidative) vs. n-type (reductive) doping. p-type is most common in energy devices (PANI, PPy, PEDOT). n-type operation is possible with specially designed backbones and dopants but is more air-sensitive [5,44].
- External (counter-ion) vs. self-doping. External dopants (acids for PANI; large anions for PPy/PEDOT) introduce counter-charges; self-doped polymers carry covalently bound ionic side groups for more stable doping [46,52].
- Secondary doping/processing. Solvent and additive treatments (e.g., DMSO/EG for PEDOT:PSS) reorganize microstructure, reduce Coulomb trapping by PSS, and raise conductivity to the 10^3^ S cm^−1^ range in thick films [51,53,54].

4. By molecular structure

- Heteroaromatic backbones: polythiophenes (incl. PEDOT), polypyrrole, polyaniline (benzenoid/quinoid forms).
- Donor–acceptor (D–A) copolymers: engineered bandgaps and energy levels for targeted transport and stability [5,46].

5. By morphology and processing state

- Dense vs. nanostructured films; electrochemically grown, solution-processed, or vapor-polymerized films. Nanofibers, core–shell coatings, and percolating hybrid networks tune tortuosity for ions/electrons and mitigate swelling-induced damage in cycling [16,55,56,57].

Implications for this review

- ICPs (PANI/PPy/PEDOT) are typically p-doped electronic or mixed conductors whose pseudocapacitive redox and high electronic conductivity underpin their roles as supercapacitor electrodes and battery binders/coatings. Their stability hinges on doping method, morphology, and swelling management [10,37,45,46].
- CPCs and hybrid films leverage percolation and interfacial engineering (CNT/graphene/MXene frameworks, oxide cores) to combine high conductivity with mechanical robustness and ion access, key for durable, high-rate energy devices [48,49,56].

## 3. Synthesis Methods, Dimensional Structures and Derivatives of Conductive Polymers

Conductive polymers (CPs) can be synthesized and processed by a broad spectrum of chemical, electrochemical and vapor-phase techniques; at the same time, their chemistry admits a large family of derivatives (doped, self-doped, grafted, copolymeric) and a wide variety of nanostructured morphologies (0D–3D). Below are provided the summarised principal synthesis routes, common strategies to produce 0D/1D/2D/3D architectures, and typical derivative chemistries used to tune electronic, ionic and mechanical properties.

### 3.1. Principal Synthesis Routes

- Chemical oxidative polymerization. In the classical bulk route, monomer (e.g., aniline, pyrrole, EDOT) is oxidized in solution by an oxidant (FeCl_3_, ammonium persulfate, etc.) to produce polymer chains and oxidized counter-ions; this method is widely used to prepare powders, films (via casting) and templated nanostructures because it is simple, scalable and tolerant to additives. Chemical oxidative routes remain the workhorse for PANI and PPy preparation [58,59].
- Electrochemical (electropolymerization). Electropolymerization deposits CP directly onto a conductive substrate by applying an oxidative potential to a monomer solution. This gives precise control of film thickness, doping state and adhesion, and is widely used for sensor and electrode coatings as well as for producing nanofibers and porous films by controlling current/potential waveforms [60].
- Vapor-phase methods (VPP/oCVD/oxidative CVD). Vapor-phase polymerization (VPP) and oxidative chemical vapor deposition (oCVD) form CP films without solvents and without PSS-type dispersants: an oxidant layer or co-vaporized oxidant initiates polymerization of monomer vapor at the substrate surface. These dry methods yield conformal, high-quality PEDOT (and other CP) films on complex or non-conductive substrates and are increasingly important for roll-to-roll and device-grade coatings [61].
- Template-assisted and interfacial polymerizations. Soft- and hard-templating (using surfactants, colloids, or sacrificial nanostructures) and interfacial polymerizations (fluid–fluid interfaces, or vapor–liquid) are used to engineer morphology (porous scaffolds, hollow spheres, nanofibers) with high surface area and tailored pore architecture for electrochemical applications [60,62].
- Green, enzymatic and plasma methods. Newer, milder oxidative systems (enzymatic oxidation, benign oxidants) and plasma polymerization approaches have been demonstrated to reduce environmental/processing footprints or to access unusual chemistries and adhesion to substrates. These approaches are less mature but growing in interest for sustainable processing [59,63].

### 3.2. Dimensional Control (0D–3D Architectures) and How They Are Made

Controlling morphology across dimensionalities is crucial because shape and size strongly influence electrical transport, ion accessibility and mechanical behaviour.

- 0D (nanoparticles, nanospheres). CP nanoparticles and nanospheres are typically produced by microemulsion or precipitation polymerization, or by fragmenting larger structures; they provide a large interfacial area and can be used as inks, additives or redox-active particles in composite electrodes [62,64].
- 1D (nanofibers, nanorods, nanotubes). One-dimensional morphologies are commonly created by templating (porous membranes, surfactant assemblies), by rapid mixing/soft-templating during chemical oxidative polymerization, or by electrospinning composite precursors followed by polymerization. 1D structures (e.g., PANI nanofibers, PPy nanotubes) improve electron percolation and shorten ion diffusion paths, giving superior rate performance in capacitive devices [58,65].
- 2D (nanosheets, thin films). Ultrathin films and nanosheets are obtained by electropolymerization, oCVD, VPP or by exfoliation of layered composites. 2D CP layers combine large lateral conductivity with tunable thickness, being particularly useful as hole-transport layers and transparent electrodes (e.g., PEDOT films from oCVD/VPP) [61,66].
- 3D (porous networks, hydrogels, foams, macroporous scaffolds). Three-dimensional CP architectures, crosslinked networks, aerogels, hydrogels and scaffolded composites are formed by templating (sacrificial templates), freeze-casting, in-situ polymerization within a porous host (e.g., foams, fabrics), or by layer-by-layer assembly. 3D CP electrodes provide high areal capacitance and mechanical robustness for flexible devices. Recent reviews summarize strategies to create mechanically compliant, ion-permeable 3D CP electrodes for supercapacitors and batteries [63,67].

### 3.3. Chemical Derivatives and Design Strategies

Tuning the polymer backbone and side chains, and creating composites, are central to optimizing CP properties:

- Doped vs. self-doped polymers. Traditional doping introduces mobile counter-ions (external dopants) to generate polarons/bipolarons; alternatively, self-doped polymers covalently attach ionic pendant groups (sulfonates, carboxylates) to the backbone to stabilise charge carriers without mobile counter-ions; this improves stability and ionic transport in some environments [68,69].
- Copolymers and grafted derivatives. Copolymerization (random, block, graft) allows combining conjugated backbones with functional side chains (ion-conducting, hydrophobic, crosslinkable) to tune solubility, film formation, mechanical toughness and energy levels for optoelectronic devices [66].
- Crosslinking and network formation. Chemical crosslinking or electrochemical crosslinking stabilises morphology against swelling and mechanical degradation during redox cycling, an important route to improve cycling stability in supercapacitors and battery coatings [63].
- Hybrid/composite strategies. Embedding CPs with 0D/1D/2D conductive fillers (carbon black, CNTs, graphene, MXenes) or inorganic oxides (TiO_2_, MnO_2_) combines the redox activity of CPs with high conductivity, mechanical support and structural stability; composites are among the most effective solutions to the cycle-life limits of pure CP electrodes [62,70].

### 3.4. Practical Considerations and Trends

Choice of synthesis method is usually governed by the target morphology, substrate, scalability and device compatibility: electropolymerization for precise lab-scale coatings, chemical oxidative polymerization for bulk powders and templated nanostructures, and vapor-phase methods (VPP/oCVD) when solvent-free, conformal films on temperature-sensitive or non-conductive substrates are required. Emerging trends include greener oxidants and enzymatic routes, self-doped backbones to improve dopant stability, and the use of additive-free vapor methods to produce device-grade PEDOT and other CP films for flexible electronics and large-area energy devices [59,61,68].

## 4. Materials: Key Conductive Polymers

### 4.1. Taxonomy and Charge Transport in Conductive Polymers

Conductive polymers (CPs) are π-conjugated macromolecules that become electronically conductive upon doping. Doping generates delocalized (bi)polaron states and shifts the Fermi level into intra-gap bands, enabling metallic-like or semiconducting transport depending on carrier density and microstructure [10,44,45,46]. Within CPs it is useful to distinguish: (i) classic intrinsically conducting polymers (ICPs) such as polyaniline (PANI), polypyrrole (PPy), and polythiophenes (e.g., PEDOT); (ii) redox-active polymers (e.g., nitroxide/quinone polymers) that store charge through localized, fast Faradaic reactions; and (iii) organic mixed ionic–electronic conductors (OMIECs), a broad class of dopable semiconducting polymers whose backbones conduct electrons while their side-chain chemistry and microstructure enable reversible ion uptake/expulsion [5,10,45,46,50]. In situ spectro-electrochemistry shows that electrochemical doping in CPs proceeds via coupled ion–electron insertion and structural rearrangements, with polarons/bipolarons forming and evolving under bias, which directly governs rate capability and stability in devices [5,45,50].

Mechanistic implications across applications:

- Supercapacitors: pseudocapacitance from fast, reversible redox along the conjugated backbone; charge is stored with counter-ion ingress/egress across nm-scale domains-hence the critical role of porosity and swelling control [10,37,46,71].
- Batteries: CPs act as (a) redox-polymer electrodes, (b) conductive binders/interlayers that form percolating electron networks and buffer volume change, and (c) organic mixed ionic–electronic conductors (OMIEC) coatings that reduce interfacial impedance and modulate SEI chemistry [50,71,72].
- Solar cells: CPs provide (a) catalytic counter-electrodes in DSSCs (triiodide reduction), (b) hole-transport layers (HTLs) with tuned work function and interfacial passivation in OPV/PSC, and (c) transparent electrodes when sufficiently conductive/optically clear [35,46,51,53,73,74].

### 4.2. Polyaniline (PANI)

Polyaniline (PANI). PANI has been widely used in energy applications due to its facile synthesis and tunable conductivity. It exists in three oxidation states—leucoemeraldine (fully reduced), emeraldine (half-oxidized), and pernigraniline (fully oxidized)—with only the emeraldine form being intrinsically conductive [75,76]. In practice, PANI is typically synthesized as the emeraldine base and then acid-doped to form the conductive emeraldine salt. Doping (protonation) introduces charge carriers along the backbone and dramatically lowers the band gap [52,77]. For example, emeraldine in its undoped form is nearly insulating (conductivity ∼10^−9^–10^−7^ S/cm), but upon acid doping, its conductivity can increase by many orders of magnitude (often 10–100 S/cm or higher) [78]. PANI is valued for being lightweight, relatively non-toxic, and thermally stable, and it offers very high pseudocapacitance arising from its reversible redox between benzenoid and quinoid units [79,80]. These attributes make PANI promising for supercapacitor electrodes and as an additive in batteries, although its cycle life and mechanical robustness can be limiting factors.

Supercapacitors: mechanisms and design. PANI delivers pseudocapacitance through the leucoemeraldine, emeraldine, and pernigraniline transitions with counter-ion uptake. Nanoscale morphologies (nanofibers) and carbon/oxide composites shorten ion/electron pathways and mitigate mechanical degradation, improving retention over 10^3^–10^4^ cycles [49,56,57]. Still, volumetric strains and dopant loss remain primary failure modes [37,81].

Batteries: mechanisms and roles. As a redox-polymer electrode, PANI provides fast kinetics but moderate capacity; more commonly, ultrathin PANI coatings/binders establish continuous electron pathways, reduce interfacial resistance, and buffer volume change (e.g., Si, LFP), improving rate and cycling [72,82].

Solar cells: mechanisms and roles: In DSSCs, PANI counter-electrodes catalyze I3– reduction via proton-coupled electron transfer, often rivaling Pt when nanoengineered; in solid-state DSSCs, PANI-based gel electrolytes/ionomers enhance interfacial contact and long-term stability [55,83,84].

**Figure 2:**
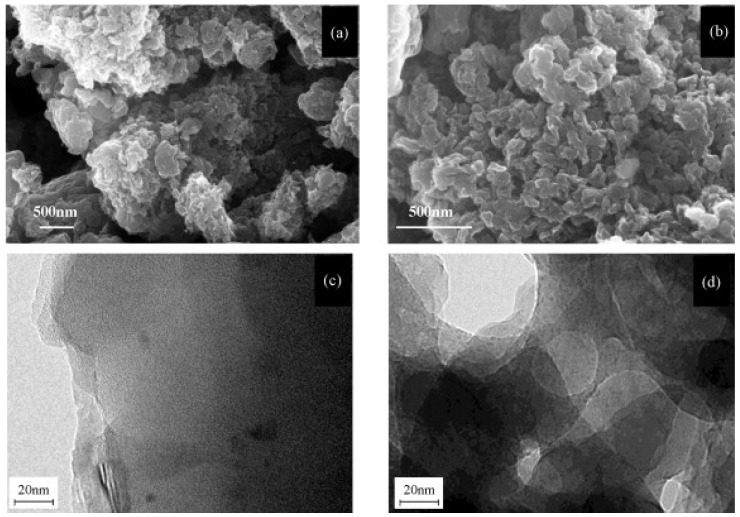
Scanning electron microscopy (SEM) images of nonporous PANI (**a**) and porous PANI (**b**), along with transmission electron microscopy (TEM) images of the nonporous (**c**) and porous (**d**) PANI samples [85]. (Permission for use is granted by Elsevier).

Designing an optimized nanostructure is essential for improving the performance of PANI-based supercapacitor electrodes. Since the morphology of PANI is heavily influenced by the synthesis technique, selecting an appropriate fabrication method is critical. Authors of [85] synthesized porous PANI using an in situ aqueous polymerization approach and evaluated its electrochemical capacitance in comparison with its nonporous counterpart. Scanning electron microscopy (SEM) images provided in Figure 2 demonstrate that the porous variant exhibited a higher density of smaller, irregularly distributed pores relative to the more uniformly structured nonporous PANI.

### 4.3. Polypyrrole (PPy)

Polypyrrole (PPy). PPy is another classical CP noted for its high conductivity and environmental stability. It typically exhibits good oxidative stability and can be synthesized easily by chemical or electrochemical oxidation of pyrrole monomers. Typical doped PPy films have conductivities in the range of tens to hundreds of S/cm. Like PANI, PPy undergoes reversible redox reactions (p-doping) that endow high capacitance [47,86]. However, PPy is intrinsically insoluble and difficult to process in solution (strong chain interactions), which complicates fabrication of large-area films [87]. Its mechanical flexibility is better than many inorganic materials, but brittleness and cycling stability remain issues. To improve processability and tailor properties, PPy has been chemically modified (e.g., N-substitution, copolymerization, or use of functional dopants). These strategies yield derivatives with improved solubility or functionality (for instance, grafting sulfonic acids can make PPy dispersible in water). In nanocomposites (with carbon nanotubes, graphene, metal oxides, etc.), PPy provides high pseudocapacitance and electrical connectivity [48,49]. PPy films deposited electrochemically can reach conductivities ~100 S/cm, and in composite electrodes PPy improves ion diffusion and cycle life [82,88,89].

- Supercapacitors: mechanisms and design. PPy stores charge by backbone redox with counter-ion motion; hierarchical PPy/carbon or PPy/oxide scaffolds improve ion transport and suppress cracking, boosting capacitance and lifetime [87,90,91,92].
- Batteries: mechanisms and roles. PPy coatings on intercalation particles (e.g., LFP) enhance percolation, reduce contact resistance, and can modulate surface chemistry; PPy networks used as conductive binders/interlayers add elasticity and conductivity in thick electrodes [5,71].
- Solar cells: mechanisms and roles. PPy counter-electrodes in DSSCs provide catalytic activity toward I3– reduction and are solution-processable, enabling low-cost alternatives to Pt; performance improves with dopant/structure control and nanocarbon hybrids [47,90].

**Figure 3:**
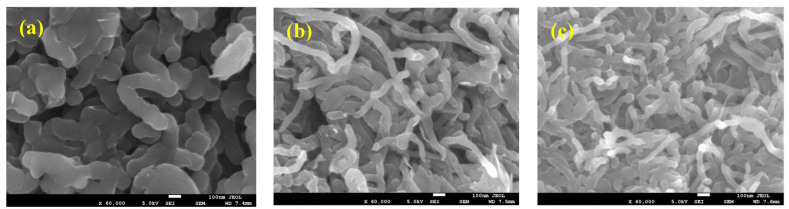
(**a**–**c**). SEM images depicting thin films of polypyrrole/multiwalled carbon nanotube (PPy/MWCNT) composites [93]. (The figure is available Open Access).

In a previous study [93], the surface morphology of the synthesized pure polypyrrole (PPy) and PPy/MWCNT composite materials was characterized using scanning electron microscopy (SEM). SEM images of the PPy/MWCNT composites are presented in Figure 3a–c. As shown in Figure 3a, the polypyrrole matrix incorporates embedded carbon nanotubes. The morphology of the resulting PPy structures—such as their shape and size—is largely influenced by the type of surfactant employed during polymerization. Specifically, PPy films or sheets can be formed using sodium dodecyl sulfate (SDS) as a surfactant and FeCl_3_ as the oxidant. Variations in synthesis parameters, such as the molar ratios of pyrrole to surfactants or oxidizing agents, can significantly affect chain bonding and structural development. Figure 3b,c display SEM images of MWCNTs coated with PPy at varying MWCNT loadings, revealing that higher MWCNT content results in a thinner PPy coating.

### 4.4. Poly(3,4-ethylenedioxythiophene) (PEDOT) and PEDOT:PSS

Poly(3,4-ethylenedioxythiophene) (PEDOT) and PEDOT:PSS. PEDOT, often used in its PSS-stabilized form, and is a highly conductive and transparent polymer widely used as an electrode or charge-transport layer. It has excellent thermal and environmental stability and can achieve conductivities of hundreds to thousands of S/cm (especially after post-treatment or secondary doping) [54]. PEDOT:PSS is dispersible in water and easily formed into thin films by solution methods. Its mechanical flexibility, low sheet resistance, and transparency make it ideal for flexible devices and transparent electrodes. For example, DMSO-treatment of PEDOT:PSS yields film conductivities above 1000 S/cm in thick films [51,53]. In many studies, PEDOT (often as the PSS complex) has demonstrated superior performance as a counter-electrode material in DSSCs and as hole-extraction layers in organic photovoltaics. Its ease of processing (spin-coating, printing, etc.) and good adhesion to various substrates further enable scalable fabrication. However, PEDOT:PSS is also quite hygroscopic (PSS is water-attracting), so stability under humidity can be an issue. Recent reviews note that PEDOT:PSS is one of the most widely used CPs precisely due to its high stability, low resistance, and ease of film formation [94,95,96].

- Supercapacitors: mechanisms and design. PEDOT stores charge through delocalized backbone redox with minimal structural disruption compared with PANI/PPy; as films/gels, it can conduct both electrons and ions, serving as an active electrode, conductive binder, or polymer electrolyte in solid-state devices [16,21,22,34].
- Batteries: mechanisms and roles. PEDOT-based binders/interlayers create percolating electronic networks and elastic interfaces, reducing impedance growth and accommodating volume change; OMIEC behavior improves Li^+^ access at interfaces.5,9,10 PEDOT coatings on high-capacity anodes and conductive binders in thick cathodes improve rate and cycling stability [50,71,72].
- Solar cells: mechanisms and roles. As HTL, PEDOT:PSS provides a high work function, surface passivation, and excellent wetting; in PSCs/OPVs, solvent or superacid treatments tune work function, reduce interfacial trap density, and improve fill factor and stability [35,51,74]. However, PSS acidity/hygroscopicity can harm perovskites/electrodes; neutralization/crosslinking and PSS-lean formulations mitigate this [51,53,54].

**Figure 4:**
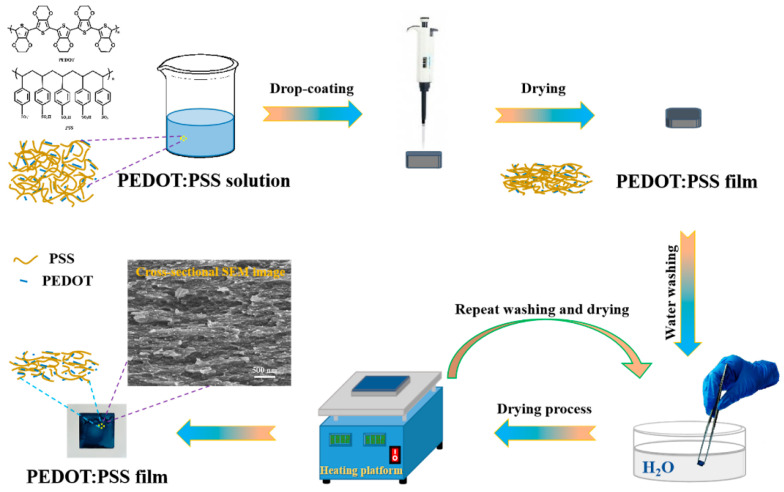
Illustration depicting the mechanism behind the formation of PEDOT:PSS films subjected to water treatment [97]. (The figure is available Open Access).

Figure 4 illustrates the schematic workflow of the water treatment applied to PEDOT:PSS films. The initial films were fabricated using a standard drop-casting technique and allowed to dry under ambient conditions. To selectively extract the PSS component and enhance the molecular ordering of the PEDOT chains, the dried films were briefly immersed in deionized water. Because of water’s tendency to dissolve the film, immersion time was restricted to 10 s, followed by an air-drying phase. This soaking and drying cycle was repeated multiple times to better assess the cumulative influence of water exposure on the film structure. The treatment process promoted the development of a porous, interconnected morphology. The efficient elimination of PSS is expected to significantly improve the electrical conductivity and thermoelectric performance of the films, as detailed in later sections.

### 4.5. Cross-Cutting Guidelines

The performance is governed not only by intrinsic conductivity but also by balancing ionic coupling, structural stability, and microstructural organization under operating conditions. Key design considerations can be summarized as follows:

(1) Match ion-coupling to task: Maximize ion accessibility (porosity/soft segments) for pseudocapacitors; in batteries, favor thin, elastic OMIEC interlayers to minimize impedance; in solar cells, minimize ion flux at HTLs to avoid drift while preserving energetics [45,53,73].

(2) Stability vs. capacitance trade-off: PANI/PPy yield high pseudocapacitance but undergo larger volumetric swings; PEDOT is more dimensionally stable but somewhat lower in specific capacitance-composites often deliver the best balance [16,21,22,24].

(3) Microstructure: Secondary doping, crystallinity, percolation, and phase purity dictate transport and durability across all three domains [51,53,74].

### 4.6. Chemical Modification and Targeted Doping

A focused discussion of chemical modifications, including halogenation (F/Cl/Br), is included because these strategies are widely used to tune a polymer’s conductivity, energy levels, stability, and interchain interactions.

- Halogenation/fluorination (F substitution). Fluorination of conjugated backbones is a widely used strategy that changes electron affinity, backbone planarity, and intermolecular interactions. Fluorinated conjugated polymers have been demonstrated to improve energy-level alignment in PVs and to stabilize backbone conformations that favor interchain π–π coupling; in thin films, this can increase charge mobility and tune surface energy for improved film formation. Recent reviews summarize rapid progress in fluorinated conjugated polymers for electronic/optoelectronic applications [98,99].
- Chlorination/bromination. Chlorination or bromination similarly modifies electronic properties and can alter chemical stability or solubility. While less exploited than fluorination for high-mobility devices, halogen substitutions have been used to tune sensor sensitivity, electrodeposition behavior, or interfacial dipoles in thin-film layers. Emerging studies are exploring the influence of heavier halogens on polymer membrane stability and selectivity, which is relevant for ion-conducting films and protective coatings [100,101].
- Counter-ion and secondary-dopant engineering. Beyond covalent substitution, the choice of dopant (PSS vs. alternative polyanions, ionic liquids, small-molecule dopants) and post-treatments (DMSO, acids, water-soak, superacid fumigation) strongly affects film conductivity and stability. For PEDOT:PSS, secondary doping and solvent treatments routinely boost conductivity by orders of magnitude and also change film morphology and hydrophobicity—directly relevant for device longevity and contact resistance [102,103].
- Crosslinking and self-doping. Introducing crosslinkable groups or self-doping side-chains (sulfonates, phosphonates) improves film robustness and minimizes dopant leaching. Self-doped or crosslinked CP thin films are attractive for harsh electrochemical environments (batteries, aqueous electrolytes) because they retain conductivity and adhesion under cycling [36,104].
- Design principle for selection. In practice, when tailoring a thin CP film for a given device, one commonly optimizes along three axes: (i) electronic conductivity (for low ESR and high power), (ii) chemical/electrochemical stability (for long cycle life), and (iii) ion transport/interfacial compatibility (for efficient charge transfer and minimal interfacial resistance). Chemical substitution (including halogenation), dopant choice, and film post-treatment are the main levers to tune these axes [9,105].

## 5. Thin-Film Fabrication Techniques

A key benefit of these materials is that they can be formed into uniform thin films or coatings by a variety of methods. In solution processing, CPs or their precursors can be spin-coated, dip-coated, or printed onto substrates, taking advantage of their solubility or dispersibility [106,107]. Chemical or electrochemical polymerization directly yields films on electrodes. For example, electrochemical deposition allows direct growth of PANI or PPy films with controllable thickness. Advanced techniques like Chemical Vapor Deposition (CVD) or oxidative vapor-phase polymerization have also been developed to coat complex shapes [108,109]. Importantly, layer-by-layer (LBL) assembly has emerged as a flexible bottom-up route to build ultrathin multilayer CP films. In LBL, alternating layers of oppositely charged polymers (or polymers with nanoparticles) are deposited, enabling precise nanoscale control over film architecture. LBL-assembled CP multilayers have been shown to produce nanostructured electrodes with improved conductivity and capacitance [56,110]. For example, stacking alternating CP and oxide layers can yield high-surface-area porous films that exhibit excellent electrochemical performance [76]. In summary, modern fabrication strategies, ranging from simple solution casting to precision LBL assembly, allow tuning the morphology, thickness, and composition of CP thin films for optimal device integration [57,111].

The performance of CP films in devices depends critically on the fabrication method, which determines film thickness, morphology, and interfacial quality [112]. Key thin-film deposition approaches include

- Solution Processing (spin/dip/spray-coating, drop-casting): Simple casting or spin-coating from polymer solutions is often used for CP:PSS and soluble derivatives. These methods yield uniform films but may leave residues or require annealing. For example, drop-cast PANI films on FTO were optimized for pH sensors https://www.mdpi.com/2073-4360/16/19/2789, accessed on 25 August 2025 [113]. Layer thickness can be tuned by solution concentration and spin speed [114]. However, pure CPs (like PPy or PANI) often require acid dopants, so their solutions must be carefully prepared. Solvent choice and drying rate affect film porosity and roughness [115]. These methods scale well for large areas, but controlling ultra-thin, pinhole-free layers can be challenging without additives or post-treatment.

**Figure 5:**
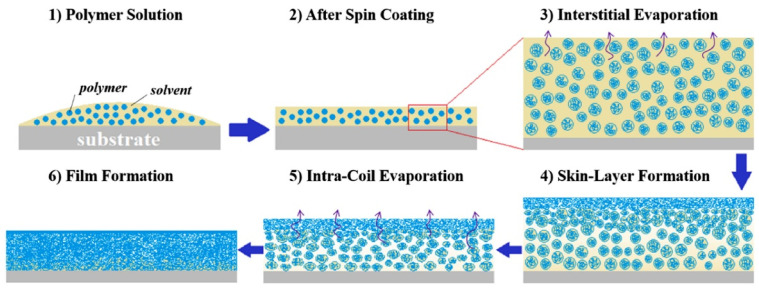
A conceptual illustration of how a film develops during the spin coating process [116]. (Permission for use is granted by Elsevier).

Understanding how film topography relates to domain size requires analyzing the fundamental stages of the spin coating process. At the outset, spinning spreads the polymer solution evenly across the substrate. When using dilute solutions (where c = c_0_ ≪ c*), solvent tends to evaporate first from the areas between polymer coils, known as interstitial evaporation, rather than from inside the coils themselves, due to differences in chemical potential [116]. As this evaporation continues, polymer coils gradually concentrate at the air–solution boundary, forming a surface layer with a polymer concentration nearing the overlap threshold (c*), as depicted in Figure 5.

**Figure 6:**
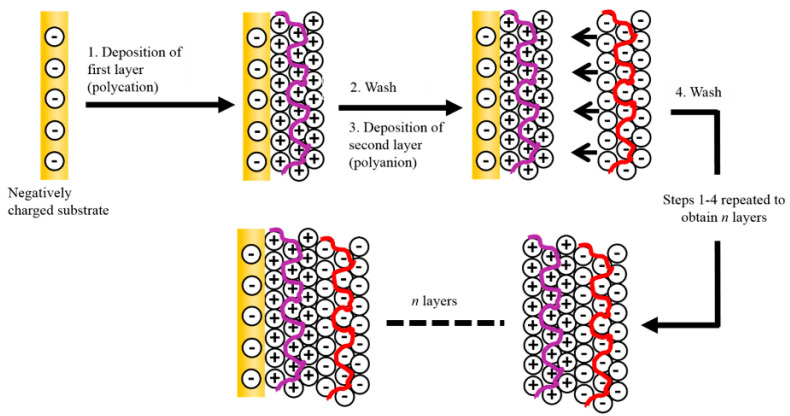
Diagrammatic representation of multilayer films constructed through the layer-by-layer (LbL) technique using electrostatic attraction: The process involves a negatively charged substrate undergoing repeated, alternating immersions in polyelectrolyte solutions, enabling the stepwise buildup of *n* distinct layers [130]. (The figure is available Open Access).

- Electrochemical Polymerization: Direct electrodeposition is widely used to grow CP films on conductive substrates [117,118]. In this method, a monomer solution with an electrolyte is used, and applying a potential or current oxidizes the monomer to initiate polymerization on the working electrode. This yields highly adherent, uniform films whose thickness can be precisely controlled by deposition time or charge https://www.mdpi.com/2073-4360/16/16/2233, accessed on 25 August 2025 [119,120]. For example, IBM’s early work produced PPy films with conductivities ~100 S/cm by electropolymerization [121]. The advantages include strong adhesion, direct deposition on the device electrode, and avoidance of residual oxidants or binders. However, electropolymerization is limited to conductive substrates (e.g., metal foils, FTO glass) and typically small areas defined by the electrode. The polymer is doped in situ by incorporating counterions during growth, but over-oxidation can degrade the polymer. Rigid process control is needed to obtain uniform films. Electrochemical methods are favored for lab-scale and sensor-type devices [45], and have been scaled in roll-to-roll processes by using moving webs as electrodes.
- Vapor-Phase Polymerization (VPP) and Oxidative CVD: Vapor-based techniques allow deposition of CP films even on non-conductive or irregular substrates. In oxidative vapor-phase polymerization (often called VPP), a thin layer of oxidant is first coated on the substrate, then exposed to monomer vapor under controlled atmosphere [55,61]. The monomer polymerizes upon contact with the oxidant layer, forming PEDOT or other CP films. This can produce uniform, smooth films over large areas, and is compatible with flexible substrates (plastic) [122]. Similarly, oxidative Chemical Vapor Deposition (oCVD) co-vaporizes monomer and oxidant in a vacuum chamber to deposit conformal polymer films [123]. oCVD has been used to coat complex 3D structures and achieve high-quality CP films for devices [124]. These methods offer precise control of film composition and purity, and avoid solvent impurities. A trade-off is the complexity of equipment and process parameters (temperature, pressure, oxidant concentration) to control polymerization kinetics [125].
- Layer-by-Layer (LbL) Assembly: Multilayer self-assembly techniques have been applied to build ultrathin CP films. In LbL deposition, alternating layers of oppositely charged materials (polyelectrolytes, CPs, nanoparticles) are sequentially adsorbed, creating nanometer-scale control of thickness. For conductive polymers, LbL allows combining CP layers with other functional materials (carbon nanotubes, enzymes, etc.) [126]. The approach is especially useful in flexible electronics and sensors because it can produce very conformal coatings on textured substrates. A recent review notes that PANI, PPy and PEDOT are the most used CPs in LbL construction [127,128]. LbL films exhibit well-controlled morphology and can integrate sensing molecules or catalysts [129]. This method is generally slow (layer-by-layer) and better suited for research or specialized thin layers rather than bulk electrode fabrication. It traditionally relies on the sequential deposition of oppositely charged polyelectrolytes, known as polyanions and polycations, onto a charged surface, whether rigid or flexible, through electrostatic interactions. The process begins by immersing the charged substrate into a solution containing a polyelectrolyte of opposite charge, allowing it to adsorb onto the surface. After this initial layer forms, the substrate is rinsed with deionized water to eliminate any loosely attached polyelectrolyte. Once the surface charge is reversed, the substrate is then immersed in a solution containing the second polyelectrolyte of opposite charge, followed by another rinsing step [130]. These alternating adsorption and rinsing cycles are repeated multiple times to achieve the desired number of layers and overall film thickness, as demonstrated in Figure 6.

- **Nanocomposite and Hybrid Films:** Embedding conductive polymers in composites or nanostructures is a major strategy to enhance film performance. For example, combining PANI or PPy with 2D materials like MXenes or graphene yields hybrid films with synergistic properties [131]. In one recent study, transparent PANI/Ti_3_C_2_ MXene films achieved high capacitance (~89 mAh/g) while retaining optical transparency [132], thanks to the MXene’s conductivity and PANI’s redox capacity. Other examples include PPy-coated CNTs or metal-oxide scaffolds, where the polymer provides pseudocapacitance and the scaffold provides conductivity and structural support [133]. Doping and polymer blending are also used, e.g., PEDOT:PSS is often mixed with conductive fillers (silver nanowires, carbon black) or treated with secondary dopants (DMSO, acids) to boost conductivity. In short, nanocomposite films exploit both the electronic properties of CP and the high surface area or conductivity of other nanomaterials, often leading to superior energy storage metrics [134,135,136].

## 6. Conductive Polymer Thin Films in Supercapacitors

### 6.1. General Charge-Storage Mechanisms in CP Films

Conductive polymers store charge via two principal mechanisms that often act together in thin-film electrodes:

1. Electric double-layer (EDL) capacitance. Charge accumulation at the polymer/electrolyte interface produces an EDL contribution similar to carbon-based materials. This contribution is generally surface-area dependent and non-faradaic [137,138].
2. Pseudocapacitance (faradaic surface or near-surface redox). CPs undergo fast, reversible redox reactions (doping/dedoping) of their conjugated backbone or pendant groups that store charge faradaically. These redox processes can be highly surface-accessible in nanostructured thin films and therefore yield high power (fast kinetics) and high capacitance per mass/area. Pseudocapacitance is the dominant mechanism for PANI and PPy in aqueous acidic electrolytes [36].

A third mechanism—ion intercalation—can occur when ions diffuse into the bulk of the polymer or into composite hosts (e.g., polymer on metal oxide, MXene, or layered carbon). Bulk intercalation increases specific capacity but may reduce rate capability and cycling stability if large volumetric changes occur [139,140].

These mechanisms explain why film thickness, porosity, morphology, and electrolyte choice must be co-optimized: thin, high-surface-area films favor surface redox and EDL behavior (fast power), while thicker/bulk intercalative electrodes can deliver higher energy at the cost of rate and cycling life [141].

### 6.2. Polyaniline (PANI) Films in Supercapacitors

Conductive polymer films excel as electrode materials for supercapacitors (particularly pseudocapacitors) because of their reversible redox chemistry. Unlike carbon electrodes, which store charge electrostatically, CPs can store additional charge via fast faradaic (redox) reactions along their backbone. For instance, PANI transitions between oxidized and reduced forms during charging, contributing large pseudocapacitive currents [142,143]. In practical devices, CP films often serve as binder-free electrodes with high specific capacitance (hundreds to thousands of F/g) when cycled in acid or polymer electrolytes. However, pure CP electrodes can suffer from volumetric swelling and mechanical degradation over many cycles [144,145,146].

PANI films (often emeraldine salt) have long been explored in supercapacitors. Early work showed PANI coatings on carbon cloth yielded specific capacitances of several hundred F/g. Modern approaches use nano-structuring and composites, e.g., PANI nanofibers or PANI/graphene hybrids. One report combined PANI with Ti_3_C_2_ MXene to create a transparent film electrode; this hybrid achieved ~89 mAh/g at 0.1 A/g with excellent ion diffusion due to the MXene network [132]. Another study found that incorporating polyaniline oligomers covalently linked to CNTs prevented polymer detachment and significantly improved cycle life [133]. Nevertheless, many PANI supercapacitors still face retention issues: the polymer can crack or lose doping over 10^3^–10^4^ cycles [78].

- Mechanism. PANI stores charge mainly via proton-coupled redox transitions between its oxidation states (leucoemeraldine, emeraldine, pernigraniline). In many practical aqueous electrolytes, the emeraldine ↔ leucoemeraldine redox yields the primary pseudocapacitive response. PANI’s pseudocapacitance is the origin of its very high reported specific capacitances [139].
- Theoretical/representative capacities. Different reports quote PANI’s theoretical capacitance/capacity in different units. Values often cited: theoretical specific capacitance on the order of several hundred to >700 F g^−1^ (or equivalently theoretical capacities up to ~290–700 mAh g^−1^ depending on which redox stoichiometry/units are used). Practically measured values are often lower due to incomplete utilization and degradation. For example, high-quality porous PANI hydrogels and composites report very large gravimetric capacitances, although capacity retention can be poor without stabilization [81].
- Enhancement strategies. Nanostructuring (nanofibers, nanotubes, porous hydrogels) to increase accessible surface area; compositing with conductive carbons, MXenes or metal-oxides to provide electronic scaffolding and reduce mechanical strain; crosslinking or covalent bonding to substrate to suppress pulverization/detachment; electrolyte engineering (polymer gels, neutral aqueous electrolytes or ionic liquids) to reduce volume changes. Recent PANI/MXene and PANI/CNT composites show large practical capacitances while improving rate and cycle life [139,147,148].

**Figure 7:**
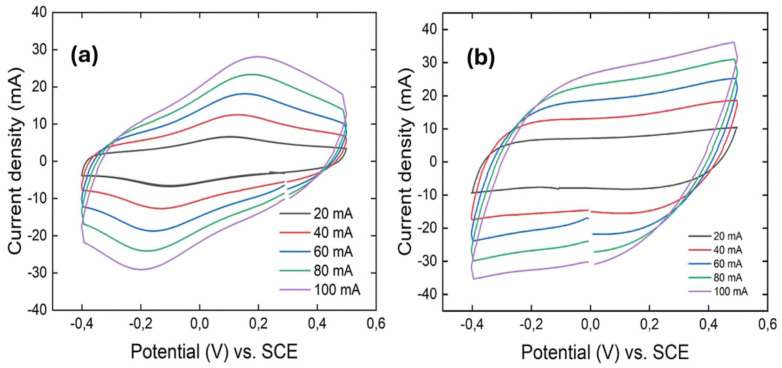
Cyclic voltammetry responses of PANI nanofibers (**a**) and three-dimensional PANI networks (**b**) [149]. (The figure is available Open Access).

Authors of the study [149] demonstrate that PANI nanofibers (Figure 7a) exhibit a quasi-rectangular cyclic voltammetry (CV) curve with a single redox pair, reflecting both electric double-layer capacitance and pseudocapacitance. The dominant redox transition corresponds to the leucoemeraldine ↔ emeraldine state, while the expected second redox peak (emeraldine ↔ pernigraniline) is absent. This is likely due to the nanofiber morphology-synthesized via a microemulsion method, which increases surface area and enhances the first redox reaction while suppressing the second due to the instability of the pernigraniline form. Additionally, the mildly acidic H_3_PO_4_/PVA-based gel electrolyte stabilizes the emeraldine state and hinders further oxidation.

In contrast, Figure 7b presents the CV curve of 3D crosslinked PANI, which shows a nearly ideal rectangular shape with no visible redox peaks, indicating dominant pseudocapacitive behavior. This is attributed to the disordered structure of the 3D network, which limits the formation of stable redox states. Although this structure offers higher conductivity and more active sites, its lower surface area restricts the extent of surface redox-based charge storage.

### 6.3. Polypyrrole (PPy) Films in Supercapacitors

Polypyrrole electrodes: PPy films similarly deliver high pseudocapacitance. For example, one PPy-based electrode synthesized by electrochemical polymerization exhibited a specific capacitance of over 300 F/g. Composite strategies have been widely adopted: PPy-coated carbon textiles, PPy/MnO_2_ core-shells, etc. A recent review highlights that integrating PPy with carbon or metal-oxide frameworks greatly enhances performance [90,121]. Notably, PPy gels and foam-like structures can accommodate volume changes, improving cycle stability. One study even reported on solid-state supercapacitors using PEDOT:PSS as the active polymer electrolyte: combining PEDOT:PSS with a high-surface-area aluminum substrate yielded devices with low equivalent series resistance (ESR) and high capacitance, outperforming conventional electrolytic capacitors in safety and stability [150].

- Mechanism. PPy exhibits fast redox doping/dedoping (p-type) leading to pseudocapacitance. In many architectures, PPy’s redox-active sites are surface-accessible, leading to good power density; however, its bulk swelling and mechanical fragility can reduce cycle life [81,151].
- Theoretical/representative capacities. PPy’s theoretical capacitance is commonly cited in the hundreds to low thousands F g^−1^ for some nanostructured composites (note these high values often reflect composite contributions). Pure PPy practical gravimetric capacitances often range from tens to several hundred F g^−1^ depending on morphology and measurement conditions [152].
- Enhancement strategies. Creating free-standing, hierarchical architectures (foams, textiles), coating conductive scaffolds (CNTs, graphene, MXene) to prevent mechanical collapse, and employing redox-active electrolytes (e.g., ZnI_2_, iodide-based) or “water-in-salt” electrolytes that stabilize cycling. Advanced PPy chemistries and electrolyte design have recently delivered flexible devices with much improved cycle life [153,154].

### 6.4. PEDOT Films in Supercapacitors

PEDOT-based electrodes: PEDOT:PSS films are often used in all-organic or solid-state capacitors. Unlike liquid electrolytes, a PEDOT:PSS gel can conduct both ions and electrons. One strategy blends PEDOT:PSS with ionic liquids or adds plasticizers to enhance ionic conductivity. In practice, PEDOT films are sometimes paired with nanoscale metal foils (Al, Ti) to form solid polymer capacitors. For example, PEDOT:PSS deposited on an etched aluminum foil yielded a capacitive device with low ESR and improved safety [155,156]. Moreover, PEDOT is often used as a conductive binder in composite electrodes: mixing PEDOT:PSS with activated carbon or MnO_2_ leads to electrodes that combine EDLC behavior (from carbon) with pseudocapacitance (from PEDOT) [157,158]. These hybrid electrodes typically show enhanced charge transport due to the polymer network, achieving higher power density and often better cyclability than neat CP films.

- Mechanism. PEDOT shows mixed ionic–electronic conduction and can contribute both a capacitive (EDL-like) and faradaic pseudocapacitive response depending on doping level and counterions. In thin-film electrodes and conductive binder roles, PEDOT often improves charge-transfer kinetics and lowers ESR [151].
- Theoretical/representative capacities. PEDOT-based electrodes usually show lower pseudocapacitance per mass than PANI/PPy but provide superior conductivity, transparency, and stability. Reported specific capacitances are commonly lower (single to low double-digit F g^−1^ for some PEDOT-only films), but hybrids (PEDOT + carbon/MXene) can achieve much higher areal or gravimetric capacitance due to the composite architecture [159].
- Enhancement strategies. Secondary doping (DMSO, EG, acids), solvent and acid post-treatment to remove excess polyanion and improve PEDOT ordering, creation of hierarchical PEDOT micro/nanofibers, and hybrid composites with carbon or MXenes to combine PEDOT conductivity with high surface area redox hosts. PEDOT also performs well as a conductive binder or electrode coating to improve device ESR and contact resistance [160].

While PEDOT:PSS is the most commonly used commercial formulation, a range of high-performance PEDOT thin films has been prepared without PSS by using alternative dopants or by dopant-free vapor/electrochemical routes. These non-PSS PEDOT films often show improved electrochemical stability, higher intrinsic conductivity or better mechanical adhesion to substrates, properties that can be advantageous for supercapacitor electrodes.

Vapor-phase polymerization (VPP) and oxidative chemical vapor deposition (oCVD) are frequently used to deposit PEDOT films in which the counter-anion is introduced from the oxidant rather than from PSS. For example, PEDOT:toluenesulfonate (PEDOT:Tos) films prepared by VPP have been shown to produce compact, high-conductivity thin films with tunable microstructure and significant pseudocapacitive behaviour when integrated into supercapacitor electrodes. Studies comparing PEDOT:Tos films produced under different VPP conditions report specific capacitances and favorable rate performance arising from the combined effect of high electronic conductivity and accessible redox sites [161].

VPP has also been used to grow PEDOT directly onto flexible cellulose or carbon scaffolds to form binder-free electrodes. For instance, vapor-phase polymerized PEDOT infiltrated into cellulose paper produced low sheet resistance electrodes that functioned simultaneously as current collectors and active materials in flexible solid-state supercapacitors, achieving good mechanical robustness and electrochemical stability under bending. Such solvent-free VPP PEDOT electrodes avoid PSS altogether and are attractive for truly all-polymer, flexible devices [162].

Other anionic dopants have been used to tune film properties and enhance charge storage. PEDOT doped with Nafion (PEDOT:Nafion) forms ionically conductive networks that improve ion transport in the electrode and have demonstrated high capacitance and excellent rate capability in several studies, indicating the potential of alternative sulfonate-type dopants to replace PSS in supercapacitor applications [163].

Recent work has also explored bulky or tailored anions and surfactant-type dopants. Directed-crystallization and deposition strategies have produced PEDOT:dodecyl sulfate (PEDOT:DS) films with enhanced crystallinity and charge transport, which are promising for applications that require a balance of high conductivity, transparency and electrochemical activity [164,165].

Finally, VPP-derived PEDOT composites (PEDOT combined with TiO_2_, graphene or other nanocarbons) have been reported, where the PEDOT matrix is formed without PSS and serves as a conformal, conductive coating over the scaffold. Such PEDOT/TiO_2_ or PEDOT/graphene films delivered specific capacitances comparable to or exceeding many PEDOT:PSS-based electrodes while benefitting from improved adhesion and reduced electrochemical swelling due to the inorganic scaffolds. These hybrid films underscore that PEDOT thin films deposited and doped via vapor or electrochemical routes (not involving PSS) are a robust and versatile family of supercapacitor electrodes [164,165].

### 6.5. Practical Performance-Improvement Strategies

From the recent literature (2022–2025), the most reproducible strategies to increase useful device performance with CP thin films include

- Nanostructuring and porosity control. Creating high-surface-area morphologies (nanofibers, porous hydrogels, 3D networks) increases pseudocapacitive active area and ion access, improving both capacitance and rate.
- Composite scaffolds (carbon, MXene, metal oxides). Adding conductive, mechanically robust scaffolds reduces volumetric stress during cycling and supplies additional EDL capacitance or intercalation sites. Notable recent advances combine CPs with Ti_3_C_2_Tx MXenes and CNT networks [166,167,168].
- Dopant/post-treatment engineering. Secondary doping, solvent treatments, partial removal of insulating counterions (e.g., PSS in PEDOT:PSS), and acid or solvent fumigation boost conductivity and improve film ordering—directly lowering ESR and increasing power [169,170,171].
- Mechanical stabilization (crosslinking, covalent anchoring). Crosslinkable monomers or covalent bonding of CPs to substrates suppress polymer detachment and cracking, improving cycling stability in PANI/PPy electrodes [172].
- Electrolyte selection and device architecture. Use of polymer gel electrolytes, neutral aqueous systems, ionic liquids, and water-in-salt electrolytes can reduce unwanted degradation pathways, widen stable potential windows, and improve cycle life. Architectures with thin CP coatings on porous supports [173,174].

## 7. Conductive Polymer Thin Films in Batteries

### 7.1. The Role of Conductive Polymer Thin Films in Batteries

In battery systems, conductive polymer films play diverse roles. Besides their use as active electrode materials (in niche polymer batteries), CPs are increasingly used as binders, conductive additives, interfacial coatings, and solid electrolytes. Their flexibility and processability make them attractive for advanced battery architectures [2,14,26]. Key points include:

- Electrode additives and binders: CPs can replace inert binders (like PVDF) and carbon black in composite electrodes, simultaneously providing electronic conductivity and mechanical cohesion. For example, PEDOT:PSS or PANI can bind electrode particles while forming conductive networks. This dual function allows reducing inactive components and increasing energy density. One review notes that embedding CP chains in thick Li-ion electrodes creates continuous ion/electron pathways, greatly improving charge transport [36]. Similarly, coating cathode particles with PPy or PEDOT improves contact and reduces interfacial resistance [90].
- Conductive coatings and layers: Thin CP films can serve as artificial solid-electrolyte interphases or protective layers. For example, PPy coatings on LiCoO_2_ cathodes have been shown to suppress parasitic reactions with liquid electrolytes and improve cycling. In Li–S batteries, PPy or PANI coatings on sulfur–carbon particles act as a conductive “sleeve” that traps polysulfides and enhances electronic connectivity [175]. These conductive coatings yield thicker, stable electrodes: one approach used a Ppy/Sulfur/PPy sandwich structure to stabilize Li–S cathodes. In all cases, the polymer film must adhere well to electrode surfaces and withstand the battery’s chemical environment.
- Flexible and solid-state batteries: For flexible or binder-free batteries, CP films can act as both current collectors and active layers. Some designs use CP-coated metal foils or fabrics as lightweight current collectors. In emerging solid-state batteries, CP-based gel electrolytes or composite membranes have been explored. For instance, PANI/PVP composite electrolytes in DSSCs (analogous systems) enhance stability by forming homogeneous polymer gel interfaces [176]. In Li batteries, CP-containing polymer electrolytes or ionomers can conduct lithium ions while providing electronic paths. Although pure CP electrolytes have limited ionic conductivity, combining them with lithium salts or ionic liquids yields quasi-solid electrolytes [177].

**Figure 8:**
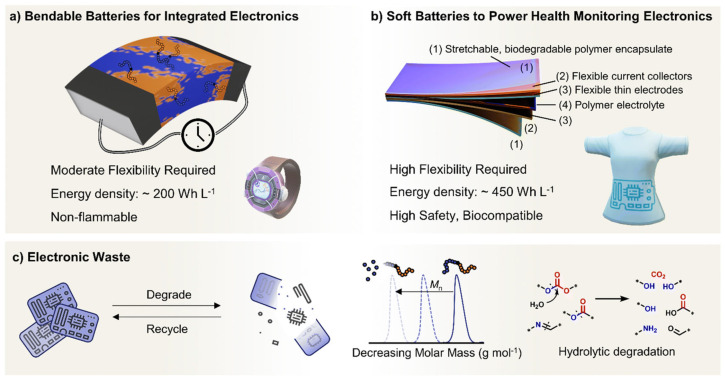
Key polymer design aspects for wearable devices: (**a**) flexible and conformal batteries suitable for powering smartwatches; (**b**) soft, body-integrated systems for direct skin contact; (**c**) disposal pathways for battery materials, highlighting depolymerization and (bio)degradation of polymers at the end of their lifecycle [178]. (The figure is available Open Access).

Designing polymers for battery applications must also address the unique demands of next-generation wearable batteries, which are central to wearable electronics. They can be classified these batteries into two main categories: (1) those embedded directly within devices such as smartwatches and foldable smartphones (Figure 8a), and (2) those worn directly on the body for health monitoring, like smart garments or eye masks (Figure 8b). Each category poses distinct requirements in terms of energy density, flexibility, and safety. As wearable devices continue to shrink and become lighter, the space available for batteries diminishes, making high energy density essential for maintaining sufficient battery life. Meeting these demands may call for advanced battery chemistries and newly tailored polymer materials beyond those previously explored [178].

- *Active polymer electrodes:* A few batteries are built entirely on redox polymers. Conjugated polymers (PANI, polythiophenes) can serve as electrode materials with energy storage capacity (albeit much lower than intercalation materials). More commonly, radical polymers (nitroxide-containing, quinones) provide fast redox at specific potentials. While not mainstream, such polymer electrodes demonstrate the versatility of CPs [179,180].

Electrically conductive functional polymers (ECFPs) in lithium-ion systems have been reviewed recently [36]. They emphasize that CP films in batteries form continuous electron networks and elastic interfaces. Such films enhance interfacial compatibility (reducing resistance and side reactions) and accommodate volume changes during cycling. For example, a thin CP coating on a Si anode can buffer the dramatic volume expansion, maintaining conductivity. Likewise, CP additives in thick electrodes create dual-conduction (electronic + ionic) networks to improve high-rate performance. The downsides are that many CPs have lower intrinsic conductivity than metals, and their dopants may leach over time.

In summary, CP thin films in batteries are valued more for mechanical and interfacial functions than as a primary active material. Their roles as conductive binders, flexible current collectors, and interfacial layers can boost battery energy/power densities and cycle life. However, the polymer components must be engineered to remain stable and conductive under battery conditions.

### 7.2. Mechanisms That Determine Battery Performance

The key mechanisms by which CPs contribute to battery performance can be summarized as follows:

1. Electronic percolation and contact resistance. As conductive binders or coatings, thin CP films form continuous electron pathways between active particles and current collectors, lowering internal resistance and improving rate capability. Optimizing film connectivity and thickness is key: too thin gives poor coverage; too thick adds inactive mass [181].
2. Interfacial chemical stabilization. CP coatings can passivate surfaces (e.g., LiCoO_2_, Si, sulfur hosts) and inhibit deleterious reactions with liquid electrolytes (electrolyte decomposition, polysulfide shuttle), thereby improving Coulombic efficiency and cycle life. For Li–S, CP interlayers or sulfur-hosting CP composites trap polysulfides and suppress shuttle losses [182,183].
3. Mechanical buffering and adhesion. For high-expansion anodes (Si), elastic CP films accommodate volume change, maintain particle contact, and reduce pulverization/detachment-leading to dramatically improved cycling when compared to traditional binders. Representative studies show PANI or PEDOT:PSS-based binders/coatings significantly improve the cycle life of Si anodes [183,184].
4. Ionic/electronic trade-offs and doping stability. Many CPs are mixed ionic–electronic conductors; their performance in batteries depends on maintaining electronic conductivity while permitting ion transport. Dopant leaching or redox-induced structural changes can degrade conductivity; crosslinking, self-doping side chains, or composite approaches are used to stabilize films [185].

### 7.3. Key Polymers in Batteries and Representative Metrics

The following examples highlight the characteristic advantages and application niches of the most widely studied conductive polymers:

- PANI (polyaniline)—Effective as conductive coatings and binders for Si and transition-metal oxide anodes. PANI coatings have been shown to pre-passivate Si and improve reversible capacity and cycle life (representative reports: reversible capacities in several hundred mAh g^−1^ with improved retention vs. uncoated Si). Stabilization strategies include covalent grafting, crosslinking and compositing with carbon networks [186,187,188].
- PPy (polypyrrole)—Widely used in Li–S cathodes and as sulfur-host composites because PPy interacts strongly with polysulfides and provides conductive networks; some PPy–S composites report very high initial capacities (>800 mAh g^−1^ at 0.1 C in lab cells) though long-term retention depends on architecture. PPy is also useful as a flexible coating for electrodes [189,190].
- PEDOT/PEDOT:PSS—Excels as conductive binder, interlayer, and current-collector alternative due to high conductivity, water-processability, and tunable work function. PEDOT:PSS composites (with PEO, crosslinkers or secondary dopants) improve electrode integrity and can act as an adsorption layer for sulfur/SEI stabilization. PEDOT:PSS-based conductive binders have been shown to improve cycle life and high-rate performance in practical electrodes [191,192].

### 7.4. Practical Strategies to Improve Battery Performance with CP Films

CPs can function simultaneously as binders, coatings, interlayers, or composite components, thereby addressing multiple performance bottlenecks at once. Representative approaches include:

1. Use CPs as multifunctional conductive binders (replace PVDF + C) to raise active fraction and maintain conductivity under cycling. Engineering waterborne PEDOT:PSS or PANI binder blends has shown improved electrode cohesion and transport properties [181].
2. Covalent anchoring/pre-passivation for Si anodes. Grafting or self-assembled monolayer-assisted PANI coatings produce more uniform lithiation and greatly extend cycle life (representative recent studies demonstrate thousands of cycles at moderate capacity retention) [193].
3. CP interlayers and sulfur hosts for Li–S. Organized CP interlayers and PPy/PANI-carbon sulfur hosts capture polysulfides and enhance Coulombic efficiency; pairing with electrolyte engineering maximizes benefit [182].
4. Composite CP + solid/polymer electrolytes for quasi-solid or all-polymer cells. Blending PEDOT:PSS with PEO or ion-conducting polymers can provide conductive networks while enabling safer, quasi-solid architectures [194,195,196].

## 8. Conductive Polymer Thin Films in Solar Cells

### 8.1. The Role of Conductive Polymer Thin Films in Solar Cells

Conductive polymers are also playing growing roles in solar photovoltaics, especially in emerging technologies. Their advantages include low cost, solution processability, and tunable energy levels. In each solar cell type, CP films have unique functions [34,197]:

- Dye-Sensitized Solar Cells (DSSCs): In DSSCs, a platinum-coated electrode normally catalyzes the redox electrolyte (I^−^/I_3_^−^). CPs like PEDOT and PANI have been successfully used as counter-electrode catalysts. For instance, PEDOT films deposited by oxidative polymerization have achieved performance comparable to Pt. One study reported a DSSC with a PEDOT CE reaching 7.88% efficiency (slightly above 7.65% for Pt) with lower charge-transfer resistance [176]. PANI and PPy CEs have also been demonstrated, sometimes with additives. A recent example doped a PANI–ZnO CE network with oxygen plasma, boosting DSSC efficiency from ~3.5% (undoped) to 6.31% by creating continuous electron pathways [198]. Conductive polymer CEs often exhibit high catalytic rates for triiodide reduction and can be fabricated by simple solution/chemical methods. Additionally, CPs have been explored as solid-state redox electrolytes or sensitizer binders in quasi-solid DSSCs, improving thermal stability by eliminating volatile liquids [84,199].
- Perovskite Solar Cells (PSCs): Perovskites commonly use organic hole-transport layers (HTLs). PEDOT:PSS is a well-known HTL in PSCs (especially for flexible devices), though its acidity can corrode some perovskites. Recent research has focused on dopant-free or cross-linked CP HTLs: for example, water-free PEDOT blends and self-doped PANI derivatives have been shown to passivate the perovskite surface and improve both efficiency and stability [200]. Conductive polymer additives inside the perovskite precursor can enhance film crystallinity and reduce defect density, leading to higher device performance. Furthermore, thin CP interlayers (e.g., a conjugated polymer film between perovskite and ITO) improve energy-level alignment and block moisture. In all cases, CP HTLs must offer high hole mobility and match the perovskite’s valence band. Polymers such as poly(3-hexylthiophene) (P3HT) or dopant-free polythiophenes have reached over 17% efficiency in CsPbI_3_ perovskites as HTLs [176]. In PSCs, CPs can also serve as transparent electrodes (replacing ITO) or electron-transport layers, though such uses are less common.

**Figure 9:**
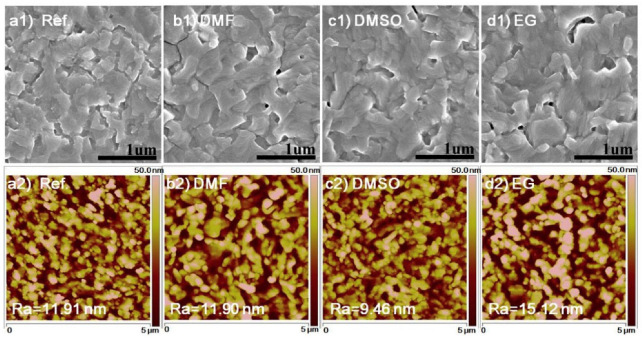
Surface characterization of perovskite layers deposited on different PEDOT:PSS substrates: SEM images (**a1**–**d1**) and corresponding AFM scans (**a2**–**d2**) for (**a**) untreated, (**b**) DMF-treated, (**c**) DMSO-treated, and (**d**) EG-treated films [201]. (Permission for use is granted by Elsevier).

In the study [201], the surface morphology of perovskite films on various PEDOT:PSS substrates was analyzed using SEM and AFM, as shown in Figure 9. Perovskite on untreated PEDOT:PSS exhibited a compact structure with an average roughness (Ra) of 11.91 nm. In contrast, films on solvent-treated PEDOT:PSS (st-PEDOT:PSS) showed larger crystal domains with similar roughness (Ra ≈ 9.5–11.5 nm). Solvent treatment removes some PSS, enriching the surface with PEDOT. The films on st-PEDOT:PSS were treated by N,N-dimethylformamide (DMF), dimethyl sulfoxide (DMSO) and ethylene glycol (EG). Those treated by EG exhibited more pinholes, which may cause charge recombination and shunting. This poor surface coverage is likely responsible for the reduced open-circuit voltage (VOC) and fill factor (FF) in these devices.

- Organic Solar Cells (OSCs): In organic photovoltaics, PEDOT:PSS is ubiquitous as the hole-extraction layer on the anode. Its high transparency and conductivity improve charge collection [202]. Similarly, CP additives in the active layer (e.g., conducting polymer-dye blends) can tune morphology and absorption. For instance, ternary OSCs often incorporate a conducting polymer as a third component to broaden absorption or stabilize the donor-acceptor phase. Conductive polymer electrodes (e.g., PEDOT on plastic) have been used to create flexible, transparent OSC modules. The success of CPs in OSCs largely stems from well-matched energy levels and simple processing.

In general, CP films in solar cells facilitate charge transport and reduce losses [203,204]. By improving interface passivation and enabling low-cost electrodes, CPs help push efficiencies upward. For example, the PEDOT CE studies and dopant-free HTM strategies mentioned above show that CP layers can match or exceed traditional materials (Pt, spiro-OMeTAD) under optimized conditions [205,206,207]. Moreover, CP-based solar cells often demonstrate better performance in low-light (indoor) conditions, expanding their application range. Nevertheless, stability under operating conditions (heat, moisture, UV) remains a concern [208,209]; many CP-PSCs and CP-OSCs see efficiency losses over time unless the polymer is carefully formulated or encapsulated [210,211,212].

### 8.2. Mechanisms and Performance Determinants

In photovoltaics CP thin films are primarily used as (i) hole-transport layers (HTLs) in organic and perovskite solar cells, (ii) transparent conductive electrodes (TCEs) or replaceable ITO (indium tin oxide) alternatives, and (iii) catalytic/counter-electrode layers in dye-sensitized solar cells (DSSCs). The defining advantages are solution processability, tunable work function, mechanical flexibility, and potential for large-area roll-to-roll manufacture [213,214,215].

1. Energy level alignment and charge extraction. As HTLs, CP films (e.g., PEDOT:PSS) adjust the interfacial energetic offset between absorber and electrode; optimal work function and low energetic disorder minimize non-radiative recombination and improve open-circuit voltage and FF. Solvent/post-treatment and dopant strategies tune PEDOT:PSS work function [216,217,218].
2. Conductivity vs. transparency trade-off for TCEs. PEDOT:PSS films can deliver low sheet resistance while retaining high transparency when post-treated or blended with conductive fillers (Ag nanowires, CNTs)—enabling flexible, ITO-free devices. The tradeoff management (thickness, secondary dopants) is critical for device efficiency [219,220,221,222].
3. Interfacial morphology and film uniformity. Smooth, pinhole-free HTLs promote uniform perovskite nucleation and reduce shunting. Solvent-treatment of PEDOT:PSS (DMSO, EG, acid fumigation) removes excess PSS and alters surface energy and roughness-leading to larger perovskite crystals and enhanced performance when properly optimized [223,224,225].
4. Chemical stability (acidity/hygroscopicity). PEDOT:PSS is somewhat acidic and hygroscopic because of PSS; this can accelerate perovskite degradation if unmodified. Strategies include neutralizing PSS, replacing PSS with alternative polyanions, crosslinking, or using interlayers to prevent direct contact. Recent HTL engineering emphasizes dopant-free and crosslinked CPs to mitigate corrosion and moisture issues [226,227,228,229].

### 8.3. Representative Polymer Systems and Studies

Their processability, electronic tunability, and compatibility with flexible substrates make them attractive alternatives or complements to inorganic charge-transport layers and electrodes. Recent studies highlight several representative applications and material strategies, including

- PEDOT:PSS as HTL/TCE. PEDOT:PSS remains the most used CP in OPVs/PSCs for HTLs and TCEs due to easy processing and tunability. Recent representative works (2023–2025) show that solvent/acid post-treatments or composite formulations (PEDOT:PSS + PEG/PEO or conductive nanowires) improve conductivity and perovskite morphology while reducing device hysteresis and improving stability. However, alternatives to PEDOT:PSS (dopant-free conjugated polymers, SAM-modified PEDOT:PSS) are emerging to tackle acidity and long-term stability concerns [230].
- PANI/PPy in DSSCs and interlayers. In DSSCs, PEDOT and PANI have been reported to replace Pt counter-electrodes with comparable catalytic activity for I_3_^−^/I^−^ redox couples in some studies; PPy also serves as cost-effective counter-electrodes and as interlayers to improve electron extraction. These CP counter-electrodes provide low cost and mechanical flexibility [231].
- Polymer additives for perovskite film quality. Tailored polymer additives, including conjugated polymers and CP fragments, act as crystal growth modulators and defect passivators in perovskite formation, leading to higher Voc and durability. JACS-Au and other 2024–2025 reviews summarize polymer strategies for boosting perovskite stability and reducing deep traps [232].

### 8.4. Practical Strategies to Improve PV Performance and Stability Using CP Films

Several strategies have been developed to optimize CP films for use as hole-transport layers (HTLs) and transparent conductive electrodes (TCEs), including

1. Work-function engineering. Use controlled doping or self-assembled monolayers (SAMs) to tune PEDOT:PSS work function for optimal hole extraction; replacing PSS or neutralizing acidity reduces corrosion of perovskites/metal contacts [233,234,235].
2. Secondary-dopant treatments for conductivity. DMSO, EG or superacid fumigation and post-treatments can increase PEDOT ordering and conductivity by orders of magnitude, enabling thinner HTLs and lower series resistance. Careful optimization avoids increased hygroscopicity or film brittleness [236,237].
3. Composite TCEs/hybrid electrodes. Blending PEDOT:PSS with Ag nanowires, CNTs or MXene layers creates hybrid transparent electrodes with low sheet resistance and mechanical flexibility-useful for large-area, flexible modules [238,239].
4. Interlayer and encapsulation strategies. Add ultrathin inert interlayers (neutral polymers, SAMs, or crosslinked CPs) between PEDOT:PSS and perovskite to prevent acid-induced degradation while maintaining good charge transfer [240,241,242,243].

## 9. Summary of Key Data in Tabular Form

### 9.1. Summary and Comparison of Synthesis Methods for Conductive Polymer Thin Films

The synthesis route chosen for conductive polymer thin films has a profound influence on their structural, morphological, and electrochemical characteristics. Different fabrication techniques offer trade-offs between control over film properties, scalability, and environmental compatibility. Table 1 summarizes the principal synthesis methods discussed in this review, outlining their advantages, disadvantages, and representative literature examples.

### 9.2. Key Properties of PANI, PPy, and PEDOT:PSS Thin Films in Energy Applications

The intrinsic material properties of conductive polymers determine their suitability for specific energy storage and conversion applications. Among the most widely studied CPs—PANI, PPy, and PEDOT:PSS—differences in conductivity, stability, and processability lead to distinct strengths and limitations in supercapacitors, batteries, and solar cells. Table 2 compares these key attributes within the context of energy-related use.

### 9.3. Representative Performance Metrics for CP-Based Devices (Recent Studies)

Performance metrics reported in the recent literature provide valuable benchmarks for evaluating the practical potential of conductive polymer–based devices. These values, while highly dependent on device architecture and test conditions, highlight the capabilities and limitations of CP thin films in supercapacitors, batteries, and solar cells. Table 3 compiles representative examples from recent studies, with emphasis on metrics directly relevant to energy applications.

## 10. Conclusions and Perspectives

### 10.1. Importance of Conductive Polymer Films in the Discussed Energy Storage Devices

The rapid evolution of energy storage and conversion technologies necessitates materials that combine high electrochemical performance with mechanical flexibility, scalability, and environmental stability. Conductive polymer (CP) thin films, particularly polyaniline (PANI), polypyrrole (PPy), and poly(3,4-ethylenedioxythiophene) (PEDOT) and its derivatives-meet these criteria by offering tunable electrical conductivity, lightweight and flexible form factors, and compatibility with diverse substrates [4,10,16,21]. Their unique combination of metallic-like conductivity and polymeric processability enables integration into supercapacitors, batteries, and solar cells, fulfilling a broad spectrum of functional requirements that inorganic materials alone often cannot match.

In supercapacitors, CP thin films deliver high pseudocapacitance through fast, reversible redox processes, enabling high power density and specific capacitance values that surpass many purely electrostatic double-layer systems [7,18,23]. Their processability allows for conformal coatings on complex architectures, improving ion accessibility and minimizing resistance [16,24]. In batteries, CPs serve multifunctional roles: as conductive binders replacing inert polymers like PVDF, as protective interfacial layers to stabilize electrode/electrolyte contact, and as active materials in redox-polymer electrodes [26,36,71]. Their elasticity and ductility are especially beneficial for high-volume-change electrodes, such as silicon anodes, where mechanical buffering significantly improves cycle life [181,183]. In solar cells, CP thin films are established as hole-transport layers (HTLs), transparent conductive electrodes (TCEs), and counter-electrodes in dye-sensitized solar cells (DSSCs) [31,34,197]. Their high optical transparency, tunable work function, and low-temperature processability facilitate roll-to-roll fabrication of lightweight, flexible photovoltaic modules [33,35]. The versatility of CPs thus lies in their ability to bridge performance and manufacturing requirements across distinct energy technologies.

### 10.2. Current Challenges

Despite their versatility, CP thin films face several well-recognized limitations that hinder their widespread commercial deployment.

Cycling stability remains a primary challenge, particularly for PANI and PPy, which undergo significant volumetric changes and dopant loss during repeated redox cycling [36,37]. This leads to cracking, loss of electrical connectivity, and gradual decline in capacitance or capacity. In supercapacitors, these mechanical and chemical instabilities can result in substantial performance degradation within 10^3^–10^4^ cycles, a figure still below the stability targets for commercial devices [81,139]. While PEDOT-based films are dimensionally more stable, their specific capacitance is typically lower than that of PANI or PPy, necessitating composite strategies for balanced performance [16,21,22].

Interfacial compatibility is another critical issue. In batteries, CP coatings must maintain intimate and stable contact with active particles while withstanding reactive electrolytes [182]. In solar cells-especially perovskite devices, the acidity and hygroscopicity of PSS in PEDOT:PSS can corrode adjacent layers, accelerate degradation, and reduce operational lifetimes [51,53,226]. Surface treatments (e.g., solvent rinsing, acid neutralization, crosslinking) have been shown to mitigate these effects but may add processing complexity [223,227].

Scalability and reproducibility are also major concerns for large-area applications. Techniques such as vapor-phase polymerization (VPP) and oxidative CVD yield high-quality, uniform CP films [61,122], but require precise control over deposition parameters, and scaling these methods for industrial throughput remains non-trivial. Solution processing (spin-coating, printing) offers greater scalability but can suffer from thickness inhomogeneity, residual dopants, and solvent-related defects [114,115]. In solar modules, large-area uniformity is paramount, as local defects can cause shunting and performance losses [201].

Finally, environmental stability, particularly under humidity, temperature fluctuations, and UV exposure-limits device lifetime. In outdoor photovoltaic modules, CP films must retain their electrical and optical properties over years of operation. However, hygroscopic dopants, UV-induced degradation of conjugated backbones, and thermal cycling can compromise performance [38,39,208]. This challenge is amplified in flexible and wearable devices, where mechanical bending and repeated strain further accelerate degradation.

### 10.3. Future Prospects

Overcoming these limitations will require a combination of molecular design, morphological control, and device engineering. Composite architectures, integrating CPs with carbon nanomaterials (CNTs, graphene), MXenes, or metal oxides, offer synergistic benefits: the inorganic or carbon phase enhances conductivity and structural stability, while the CP phase contributes redox activity and interfacial adhesion [48,49,56,166]. Such hybrid films have already demonstrated improved cycle life and mechanical resilience in both supercapacitors and batteries [168,172].

Chemical modification strategies, such as self-doping, covalent crosslinking, and side-chain engineering, can improve doping stability and reduce volumetric changes [68,69,104]. In PEDOT systems, replacing PSS with less hygroscopic polyanions, or producing PSS-free films via VPP or oCVD, has shown promise for improving both conductivity and environmental stability [51,54,162]. In PANI and PPy, grafting to substrates or embedding in flexible scaffolds reduces detachment and mechanical failure [172,183].

For large-scale manufacturing, the development of roll-to-roll compatible deposition techniques that deliver uniform films with controlled thickness and morphology will be essential. Ink formulations with optimized rheology for printing, combined with post-treatment protocols (e.g., solvent vapor annealing, dopant exchange), can bring lab-scale performance closer to industrial viability [33,114,115].

In terms of lifetime and durability, protective encapsulation, environmental barrier coatings, and intrinsic stability improvements will be necessary for outdoor and wearable applications. In solar cells, for instance, CP HTLs could be engineered with hydrophobic or crosslinked backbones to resist moisture ingress and thermal stress [226,227]. Similarly, in electrochemical devices, neutral electrolytes and gel systems that minimize chemical stress on CP films could extend operational lifetimes [36,139].

Looking forward, the convergence of green chemistry with CP film synthesis-employing low-toxicity monomers, aqueous or solvent-free processes, and recyclable materials-could align these technologies with sustainability goals [59,63]. Additionally, the inherent compatibility of CPs with flexible substrates positions them as key enablers for next-generation wearable energy systems, where integration of supercapacitors, batteries, and photovoltaic modules into a single conformal platform will demand multifunctional, flexible, and stable electrode materials [178].

In conclusion, while conductive polymer thin films have already proven their utility in diverse energy devices, achieving their full potential will require coordinated advances in materials chemistry, processing technology, and device architecture. By addressing the interrelated challenges of stability, scalability, and environmental durability, CP-based thin films could play a pivotal role in the commercialization of high-performance, sustainable, and flexible energy storage and conversion systems.

## Figures and Tables

**Table 1:** Summary and comparison of synthesis methods for conductive polymer thin films.

Synthesis Method	Description	Advantages	Disadvantages	References
Chemical Oxidative Polymerization	Oxidation of monomers (e.g., aniline, pyrrole, EDOT) in solution using oxidants such as FeCl_3_ or ammonium persulfate to produce polymer powders, films, or nanostructures.	Simple, scalable, tolerant to additives; suitable for bulk powders and templated morphologies; compatible with diverse monomers.	Limited control over film thickness and morphology; may leave residual oxidants; film adhesion to substrates often poor without post-treatment.	[58,59]
Electrochemical Polymerization (Electropolymerization)	Direct deposition of CP onto conductive substrates by applying oxidative potential in a monomer solution.	Precise control of film thickness, doping state, and morphology; strong adhesion; can create porous or nanostructured films.	Limited to conductive substrates; small-area deposition unless scaled in specialized setups; risk of over-oxidation degrading polymer.	[45,60,117,118]
Vapor-Phase Polymerization (VPP)/Oxidative CVD (oCVD)	Polymerization from monomer vapor onto a substrate coated with oxidant (VPP) or via co-vaporization of monomer and oxidant (oCVD).	Solvent-free; conformal, uniform coatings on complex or non-conductive substrates; high purity; tunable microstructure.	Requires precise control of temperature/oxidant; specialized equipment; slower throughput for thick films.	[55,61,122,123,124]
Template-Assisted & Interfacial Polymerization	Polymerization in confined spaces (soft/hard templates, interfaces) to yield specific morphologies such as nanofibers, hollow spheres, or porous films.	Enables control over pore size and shape; high surface area; improved ion accessibility in electrochemical devices.	Template removal steps may be required; potential contamination from template residues; more complex processing.	[60,62]
Layer-by-Layer (LbL) Assembly	Sequential adsorption of oppositely charged CPs/polyelectrolytes to build multilayer films with nanometer-scale thickness control.	High precision over thickness and composition; can incorporate multiple functional components; good conformality.	Slow deposition; less suited for bulk electrodes; requires multiple washing steps; sensitive to solution conditions.	[56,110,126,127,128]
Green/Enzymatic/Plasma Methods	Use of mild oxidants, enzymatic catalysts, or plasma processes to form CP films.	Environmentally friendly; can improve adhesion to substrates; enables unusual chemistries.	Less mature; often lower yield; limited control over large-area uniformity.	[59,63]
Nanocomposite/Hybrid Film Fabrication	Incorporation of CP into a conductive scaffold (e.g., CNTs, graphene, MXenes, oxides) via in situ polymerization or blending.	Combines high conductivity and mechanical strength of scaffold with redox activity of CP; enhances stability and performance.	More complex processing; may require surface modification for compatibility; possible phase segregation.	[131,132,133,134,135,136]

**Table 2:** Key properties of PANI, PPy, and PEDOT:PSS thin films in energy applications.

Polymer	Typical Conductivity	Stability in Energy Devices	Scalability/Processability	References
Polyaniline (PANI)	Undoped: ~10^−9^–10^−7^ S/cm; Acid-doped: 10–100 S/cm or higher [52,77,78].	High pseudocapacitance but prone to volumetric changes, dopant loss, and mechanical degradation over 10^3^–10^4^ cycles in supercapacitors [37,81]. Stability in batteries improved by nanostructuring, composites, and coatings [72,82].	Scalable via chemical oxidative polymerization; moderate processability; insoluble in most solvents; morphology highly dependent on synthesis route [58,59].	[37,52,58,72,77,78,79,80,81,82]
Polypyrrole (PPy)	Doped films: tens to hundreds of S/cm [47,86,87].	Good oxidative stability; suffers from brittleness and swelling during cycling, reducing lifetime [81,87]. Stability improved via composites with carbon, oxides, or flexible scaffolds [90,91,92].	Easily synthesized chemically or electrochemically; poor intrinsic solubility limits some processing; can be deposited conformally via electropolymerization [45,60].	[45,47,81,86,87,88,89,90,91,92]
PEDOT:PSS	Commercial films: ~1 S/cm; after secondary doping/post-treatment (e.g., DMSO, EG), >1000 S/cm achievable [51,53,54].	Dimensionally stable under cycling; PSS component is hygroscopic and acidic, which can degrade adjacent layers (e.g., perovskites) [51,53,226]. Modified or PSS-free formulations improve environmental stability [162].	Excellent aqueous processability; compatible with spin-coating, printing, and roll-to-roll methods; large-area uniform films achievable [33,35,51].	[33,35,51,53,54,162,226]

**Table 3:** Representative performance metrics for CP-based devices.

Device/Material (Configuration)	Reported Metric (Test Condition)	Notes/Relevance	References
Transparent PANI/Ti_3_C_2_ (MXene) film supercapacitor	Specific capacity ≈ 89 mAh g^−1^ (at 0.1 A g^−1^; transparent hybrid electrode).	Shows viability of PANI–MXene hybrids for transparent, high-capacity thin-film supercaps—aligns with examples in the review.	[132,244]
PPy/carbon-based composites (e.g., PPy/C-dots, PPy/MXene supercapacitor	Specific capacitance up to ~676 F g^−1^ (composite electrodes, measured at given current densities in respective papers).	High values typically from nanostructured composites; demonstrates how composites boost practical capacitance and rate.	[1,245]
PEDOT: Nafion/PEDOT:PSS —all-solid/thin-film supercapacitors	Areal capacitance ~22 mF cm^−2^ (PEDOT/Nafion film at 10 mV s^−1^); other PEDOT:PSS-based thin films report mF cm^−2^ to F g^−1^ range depending on thickness and treatment.	Highlights PEDOT formulations acting as both active electrode and current collector in thin, flexible devices.	[21,163]
PANI-grafted Si anode (self-assembled monolayer + PANI) —Li-ion battery	Reversible capacity ~510 mAh g^−1^ after 2000 cycles (improved cycling stability reported in surface-grafted PANI–Si systems).	Emphasizes CP coatings/binders’ role in mechanical buffering and long-term cycling of high-strain anodes.	[193,246].
PANI/metal-oxide or other CP-coated anodes—Li-ion battery	Examples report capacities from ~600 to >1500 mAh g^−1^ (depending on host material, architecture; some PANI-coated Si or metal-oxide hybrids show >1000 mAh g^−1^ in lab cells).	Shows the range achievable when CPs are used as protective/conductive coatings or part of hybrid active materials.	[247,248]
Conductive polymer (PEDOT:PSS)/Si hybrid solar cell	Power conversion efficiency >12% (e.g., EG-modified PEDOT:PSS on planar Si, reported >12% in hybrid devices).	Demonstrates PEDOT:PSS as an effective HTL/contact layer for flexible/hybrid photovoltaics when optimized.	[249]
MXene-embedded PEDOT (HTM)—perovskite/PV	Reported PCE up to ~25.5% (MXene-embedded PEDOT HTM demonstrated record-level PCE in the cited work).	Example showing how CP + 2D conductive fillers can push device efficiency when used as HTL/interlayer. High PCEs typically require careful optimization and are architecture-dependent.	[249]

## Data Availability

Data are contained within the article.

## List of Abbreviations

2D	Two-dimensional
3D	Three-dimensional
AFM	Atomic Force Microscopy
CE	Counter-electrode
CNT	Carbon Nanotube(s)
CP	Conductive Polymer(s)
CP:PSS	Conductive Polymer:Poly(styrenesulfonate)
CV	Cyclic Voltammetry
CVD	Chemical Vapor Deposition
DMF	N,N-Dimethylformamide
DMSO	Dimethyl Sulfoxide
DSSC	Dye-Sensitized Solar Cell
EDL	Electric Double Layer
EDLC	Electric Double-Layer Capacitor
EDOT	3,4-Ethylenedioxythiophene
EG	Ethylene Glycol
ESR	Equivalent Series Resistance
FF	Fill Factor
FTO	Fluorine-Doped Tin Oxide
HTL	Hole-Transport Layer
HTM	Hole-Transport Material
IBM	International Business Machines Corporation
ICP	Intrinsically Conducting Polymer(s)
ITO	Indium Tin Oxide
JACS	Journal of the American Chemical Society
LBL	Layer-by-Layer
LFP	Lithium Iron Phosphate
MWCNT	Multi-Walled Carbon Nanotube(s)
oCVD	Oxidative Chemical Vapor Deposition
OMIEC	Organic Mixed Ionic–Electronic Conductor
OPV	Organic Photovoltaic
OSC	Organic Solar Cell
PANI	Polyaniline
PEDOT	Poly(3,4-ethylenedioxythiophene)
PEDOT:DS	Poly(3,4-ethylenedioxythiophene):Dodecyl Sulfate
PEDOT:PSS	Poly(3,4-ethylenedioxythiophene):Poly(styrenesulfonate)
PEG	Poly(ethylene glycol)
PEO	Poly(ethylene oxide)
PET	Poly(ethylene terephthalate)
PSC	Perovskite Solar Cell
PSS	Poly(styrenesulfonate)
PV	Photovoltaic
PVA	Poly(vinyl alcohol)
PVDF	Poly(vinylidene fluoride)
PVP	Poly(vinylpyrrolidone)
SAM	Self-Assembled Monolayer
SDS	Sodium Dodecyl Sulfate
SEI	Solid–Electrolyte Interphase
SEM	Scanning Electron Microscopy
TCE	Transparent Conductive Electrode
TEM	Transmission Electron Microscopy
UV	Ultraviolet
